# Phylogenetic signal in the acoustic parameters of the advertisement calls of four clades of anurans

**DOI:** 10.1186/1471-2148-13-134

**Published:** 2013-07-01

**Authors:** Bruno Gingras, Elmira Mohandesan, Drasko Boko, W Tecumseh Fitch

**Affiliations:** 1 Department of Cognitive Biology, Faculty of Life Sciences, University of Vienna, Althanstrasse 14, Vienna A-1090, Austria; 2 Institute of Population Genetics, University of Veterinary Medicine of Vienna, Veterinärplatz 1, Vienna A-1210, Austria

**Keywords:** Anurans, Molecular phylogeny, Phylogenetic signal, Acoustics, Advertisement calls, Mitochondrial DNA, 12 S, Rhodopsin, Bufo, Hylinae, Leptodactylus, Rana

## Abstract

**Background:**

Anuran vocalizations, especially their advertisement calls, are largely species-specific and can be used to identify taxonomic affiliations. Because anurans are not vocal learners, their vocalizations are generally assumed to have a strong genetic component. This suggests that the degree of similarity between advertisement calls may be related to large-scale phylogenetic relationships. To test this hypothesis, advertisement calls from 90 species belonging to four large clades (*Bufo*, Hylinae, *Leptodactylus*, and *Rana*) were analyzed. Phylogenetic distances were estimated based on the DNA sequences of the 12S mitochondrial ribosomal RNA gene, and, for a subset of 49 species, on the rhodopsin gene. Mean values for five acoustic parameters (coefficient of variation of root-mean-square amplitude, dominant frequency, spectral flux, spectral irregularity, and spectral flatness) were computed for each species. We then tested for phylogenetic signal on the body-size-corrected residuals of these five parameters, using three statistical tests (Moran’s I, Mantel, and Blomberg’s *K*) and three models of genetic distance (pairwise distances, Abouheif’s proximities, and the variance-covariance matrix derived from the phylogenetic tree).

**Results:**

A significant phylogenetic signal was detected for most acoustic parameters on the 12S dataset, across statistical tests and genetic distance models, both for the entire sample of 90 species and within clades in several cases. A further analysis on a subset of 49 species using genetic distances derived from rhodopsin and from 12S broadly confirmed the results obtained on the larger sample, indicating that the phylogenetic signals observed in these acoustic parameters can be detected using a variety of genetic distance models derived either from a variable mitochondrial sequence or from a conserved nuclear gene.

**Conclusions:**

We found a robust relationship, in a large number of species, between anuran phylogenetic relatedness and acoustic similarity in the advertisement calls in a taxon with no evidence for vocal learning, even after correcting for the effect of body size. This finding, covering a broad sample of species whose vocalizations are fairly diverse, indicates that the intense selection on certain call characteristics observed in many anurans does not eliminate all acoustic indicators of relatedness. Our approach could potentially be applied to other vocal taxa.

## Background

Although rich and varied, the vocalizations of anurans (frogs and toads) are relatively fixed and show no evidence of vocal learning [1,2], and are thus presumably mostly genetically determined. Some basal level of general auditory stimulation may be necessary for the neural development of species-typical advertisement calls [3], but call structure itself appears to be highly stereotyped within a species. Moreover, anuran vocalizations, especially their advertisement calls, differ considerably across species and can often be used reliably to determine species [4,5]. This leads straightforwardly to the hypothesis, inspired by Blair [6], that the degree of similarity between advertisement calls in anurans should be related to large-scale phylogenetic relationships, and that species that are evolutionarily distant would be expected, on average, to display vocalizations that are more dissimilar than species that are more closely related. However, a recent study by Tobias *et al*. [7] found only a weak phylogenetic signal in vocalizations of African clawed frogs, concluding that rapid evolution and frequent homoplasy can quickly erase acoustic indicators of phylogeny.

Detailed comparisons between differences in calling behavior and phylogenetic distances in vertebrates, involving up to 15 anuran species [8-10], 6 species of crested gibbons [11], and 11 deer species [12] can be found in the literature. However, these studies generally involved a small number of closely related species, with few exceptions such as a broader comparison involving 21 species of Bufonidae and Hylidae [13]. There are, to our knowledge, no large-scale studies investigating the link between the acoustic similarity among advertisement calls and the phylogenetic distance on a large number of species representative of the broad geographic and taxonomic distribution of anurans. Moreover, previous studies typically relied upon specific acoustic features that were customized to the vocalizations of a particular clade, thereby restricting their applicability to a limited range of species.

The current study aimed to fill these lacunae, by comparing acoustic similarity and genetic distance for 90 species of anurans belonging to four clades with a wide geographic distribution: *Bufo*, Hylinae, *Leptodactylus*, and *Rana*. More precisely, we sought to find a set of low-level acoustic parameters applicable to distant clades that display a broad range of vocalization types (the expression “low-level acoustic parameters” refers to parameters that can be reliably extracted algorithmically in a variety of acoustic signals, such as spectral flux or dominant frequency). We then assessed the degree of autocorrelation between these acoustic parameters and phylogenetic dissimilarities to estimate the extent to which differences between call acoustics are linked to genetic divergence among clades.

This investigation is relevant to larger issues relating to the relative influence of various selective pressures on vocalization characteristics. If, in a taxon of non-vocal learners such as anurans, similarities in certain acoustic parameters were consistently related to phylogenetic distances, it would suggest that phylogenetic constraints are an important factor in determining those characteristics. On the other hand, when sexual selection is the main evolutionary force shaping vocalization parameters in anurans, the affected parameters might be expected to exhibit rapid, runaway-style selection, and the link between phylogenetic relatedness and acoustic similarity should be tenuous, except for closely-related species [14]. Hence, our central aim is to understand whether some call parameters change slowly enough to retain a reliable signal of phylogeny across a broad range of clades and species.

To estimate phylogenetic distances, we chose the 12S rRNA region of the mitochondrial DNA (mtDNA), a region for which complete (or nearly complete) sequences are available for a large number of anurans and which has already been used to analyze relationships among hyloid frogs [15] and Malagasy reed frogs [8]. Although some authors have questioned the suitability of mitochondrial DNA for phylogenetic inferences [16,17], the extensive use of 12S in anuran phylogenetic studies [18,19] means that sequences are available for numerous species, and suggests that it is appropriate for our purposes. However, a potential issue with using 12S mtDNA is that this gene is prone to rapidly accumulate mutations, thus possibly leading to saturation in nucleotide substitutions, which would potentially decrease the phylogenetic information contained in the dataset [20]. Because genetic divergences in shallower relationships (such as intra-clade comparisons) could be expected to outweigh more distant relationships (such as inter-clade comparisons) in the case of a saturated gene, this could theoretically bias our analysis towards short-range relationships. We addressed this issue in two ways. First, we conducted a saturation test on the 12S mtDNA sequences to assess the degree of saturation present in our dataset. Second, we compared the results obtained using 12S mtDNA sequences to those obtained with the first exon of the rhodopsin gene, a nuclear gene, for a subset of 49 species for which both sequences were available. Nuclear protein-coding single-copy genes such as the rhodopsin gene have been shown to outperform mitochondrial sequences when analyzing deeper genetic divergences [21,22]. Note that we do not expect 12S, rhodopsin, or other genes traditionally selected for phylogenetic analysis, to play any direct causal role in vocal production: these genes simply serve as proxies for overall phylogenetic distance. At present, the direct genetic determinants of acoustic dissimilarities in the vocalizations of any vertebrate species remain to be identified.

The acoustic properties of advertisement calls were quantified using mean values computed from a series of low-level acoustic parameters following the procedure previously developed for anurans in [23]. The five parameters that this previous study showed to be most independent and informative were used: coefficient of variation of root-mean-square amplitude (CVA), dominant frequency (DF), spectral flux (SF), spectral irregularity (SI), and spectral flatness or tonality (TON). CVA refers to the standard deviation of the root mean square of the amplitude, divided by the true mean (note that, as a coefficient of variation, CVA is a unit-free measure and is thus independent of the absolute intensity of the sound or of the distance from the microphone), whereas DF (in Hz) represents the single frequency of maximal amplitude in the spectrum. SI is defined as the sum of the square of the differences in amplitude between adjoining partials [24]. SF is a time-varying descriptor that corresponds to the Euclidean distance between two spectra. Lastly, TON is computed as the ratio between the geometric mean and the arithmetic mean of the power spectrum [25], with lower values representing more ‘spiky’ spectra, indicating the presence of strong partials (but not necessarily integer-multiple harmonics).

Crucially, these parameters can be measured from a very wide variety of sounds, unlike some traditional measures such as trill rate that may not be applicable to all species or vocalizations. Moreover, these parameters refer to well-characterized acoustic properties of the vocalizations that can be directly and automatically derived from recordings. These parameters correspond for the most part to spectral features, and global temporal patterning features are not considered in our analysis. However, CVA and SF capture local aspects of temporal variability, and are thus best characterized as spectro-temporal quantities.

The acoustic database for this study is derived entirely from published, commercially-available digital collections on CDs (listed in Additional file 1). The species in our sample were originally assigned to one of four genera on the basis of their identification by the recordists: *Bufo*, *Hyla*, *Leptodactylus*, and *Rana*. Several of the recordings originally assigned to *Hyla* species on these recordings have subsequently been reclassified to other genera, but remain within the Hylinae subfamily, a monophyletic clade. Here, we follow the nomenclature used by Pyron and Wiens [19], which is more conservative than Frost’s *Amphibian Species of the World* website [26], especially regarding *Bufo* and *Rana* which mostly retain the composition that they had prior to Frost *et al.*[18]. However, given that the generic content of several anuran families remains in flux, we avoid referring specifically to “genera” or “subfamilies”, and will use the generic term “clades” to refer to our four taxonomic groupings for the remainder of the article. In any case, our analysis techniques use genetic distance as a proxy for phylogeny, and are thus robust to changes in systematic nomenclature.

Because body size imposes severe constraints on vocal signals, it is often closely related with the acoustical features of animal vocalizations. Indeed, an inverse relationship between body size and call frequency has been documented in numerous species of anurans (reviewed in [1,27,28]). In the case of the parameters selected for our analysis, both DF and TON were shown in a previous study to be inversely correlated with snout-vent length (SVL, a proxy for body size) in a sample of 136 species belonging to the four clades analyzed in the present study [29]. Furthermore, body size is generally strongly autocorrelated with genetic distance (e.g., [30,31]). It is thus necessary to partial out the relationship between body size and acoustic parameters prior to conducting a phylogenetic signal analysis. Here, we corrected for the effect of body size by first regressing the acoustic parameters on SVL using phylogenetic generalized least squares regression (PGLS) [32,33], and then evaluating the autocorrelation between the regressed residuals and the genetic distances. PGLS regression was used instead of ordinary least squares regression because ignoring phylogeny in the size-correction procedure can lead to spurious results in the subsequent phylogenetic signal analysis [34,35], and the PGLS method has been shown to be relatively robust to phylogenetic tree misspecification [36].

We tested for the presence of a phylogenetic signal in the size-corrected residuals of the acoustic parameters by applying three different statistical methods: Moran’s I test [37], Blomberg’s *K*[31], and the Mantel test [38]. Both Moran’s and Mantel tests are general procedures for testing for spatial or genetic autocorrelation [39], whereas Blomberg’s *K* is a descriptive statistic based on a Brownian (random walk) model of trait evolution (BM), with a value of 1 corresponding to the degree of trait similarity expected under BM. A *K* of 0 indicates phylogenetic independence, whereas a *K* > 1 implies trait similarity greater than expected under BM [31]. In contrast to Moran’s I and Blomberg’s *K*, which are suitable for univariate phenotypic traits, the Mantel test can be used to test for a correlation between a multivariate trait (corresponding for instance to a subset of the acoustic parameters examined here) and a distance matrix, and its power to detect a phylogenetic signal increases substantially with the number of traits [40]. Here, trait distances were obtained by computing Euclidean distances on the standardized size-corrected residuals, and the best-fitting multivariate trait distance model was determined by a stepwise forward procedure (details given in the Methods section).

Although Moran’s I, Blomberg’s *K*, and the Mantel tests are closely related mathematically [41,42], their performance depends heavily on the choice of genetic distance model [42]. For this reason, we used three different models of genetic distance. The first model, based on the pairwise genetic distance between sequences, does not take into account phylogenetic relationships and can thus be seen as ‘phylogenetically naïve’. Distance matrices based on this model were used with both Moran’s and Mantel tests. The second model was based on Abouheif’s matrix of phylogenetic proximities [43,44], which has been shown to be a powerful alternative to Blomberg’s *K*[45], especially for unresolved trees or when branch lengths are not accurate [42]. Distances matrices based on this model were also tested with both Moran’s and Mantel tests. Finally, the genetic distance model used in Blomberg’s *K* is a variance-covariance matrix derived from the phylogenetic tree, where the covariance corresponds to the branch length from the root to the most recent common ancestor. Unlike the previous distance matrices that are not based on an explicit evolutionary model [45], the variance-covariance matrix is based on the BM model. All tests were conducted both on the entire sample of 90 species, and on each individual clade.

## Results

### Testing for saturation in nucleotide substitutions in 12S mtDNA sequences

**Figure 1:**
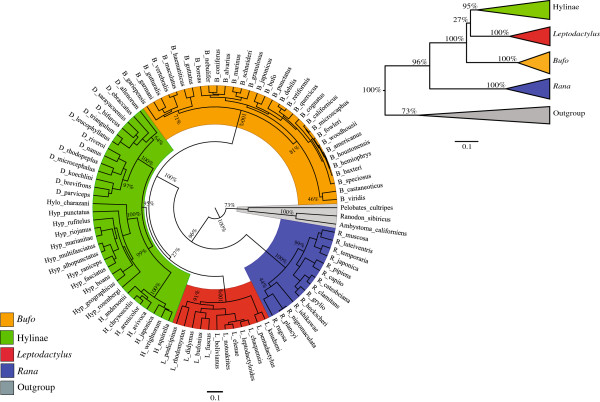
**Polar Bayesian phylogenetic tree under the GTR+Γ+I evolution model based on 12S mtDNA sequences from 90 species belonging to four clades of anurans *(Bufo, *Hylinae, *Leptodactylus *and, *Rana).*** Bayesian posterior supports for major nodes are shown. The scale bar indicates a branch length equivalent to 0.1 nucleotide substitutions per site. Abbreviations: B: *Bufo*; D: *Dendropsophus*; H: *Hyla*; Hylo: *Hyloscirtus*; Hyp: *Hypsiboas*; L: *Leptodactylus*; R: *Rana*.

Substitution saturation could potentially decrease the phylogenetic information contained in our 12S mtDNA dataset. In order to assess the degree of substitution saturation in our dataset, we used a substitution saturation test implemented in DAMBE (Data Analysis in Molecular Biology and Evolution) [46,47]. This test computes a saturation index, which is compared to a critical value determined for symmetrical and extremely asymmetrical tree topologies. The saturation index was significantly lower than the critical value when performing the analysis on fully resolved sites (P < 0.001 for both topologies) and on all sites for a symmetrical topology (P < 0.001), indicating little or no saturation. However, the saturation index did not significantly differ from the critical value when performing the analysis on all sites for an extremely asymmetrical tree topology (P = 0.281), meaning that we cannot exclude the possibility of saturation for such a topology, which remains an unlikely one in any event [47]. Given that the phylogenetic tree derived from the 12S mtDNA dataset clearly does not correspond to an extremely asymmetrical topology (Figure 1), we concluded, on the basis of these results, that saturation was likely to be minimal in our dataset (detailed results of the saturation test are provided in Additional file 2).

### Testing for phylogenetic signal in acoustic parameters based on 12S mtDNA

Four acoustic parameters (DF, SF, SI, and TON), as well as SVL, were log-transformed to achieve normality (the values of the acoustic parameters computed for each recording, as well as the SVL values obtained from the literature, are listed in Additional file 3). All subsequent analyses were conducted on the log-transformed variables. All acoustic parameters (CVA, logDF, logSF, logSI, and logTON) were then phylogenetically regressed on logSVL using the PGLS method, to partial out the effect of body size [34]. Separate regressions were conducted for each clade in the case of intra-clade analyses to account for the fact that the allometric relationship between body size and acoustic parameters may vary across clades [29].

Table 1 summarizes the results obtained for all tests on the size-corrected residuals of the acoustic parameters. Across the entire sample of 90 species, significant values, indicating the presence of a phylogenetic signal, were observed for all 5 acoustic parameters using Moran’s test on either the pairwise genetic distances or on Abouheif’s proximities, and with all parameters except for logSI in the case of Blomberg’s *K*. In contrast, the Mantel test revealed a significant correlation with pairwise distances only for logDF and logSI, and no multivariate trait exhibited a higher correlation than logDF. The discrepancy between the results obtained with Moran’s I and the Mantel test on the pairwise distances is presumably due to the fact that Moran’s I tends to be affected by extreme values [48], whereas the Mantel test, which is nearly equivalent to Geary’s C (another measure of spatial autocorrelation, see [49]) in the case of Euclidean distances computed from univariate data (see [39], Appendix 1), tends to be more sensitive to local autocorrelation (Geary’s C also reached significance only for logDF and logSI on the pairwise distances). However, Mantel tests on Abouheif’s proximities yielded significant correlations for all 5 acoustic parameters, in line with Moran’s test, and a multivariate trait based on the Euclidean distances computed from CVA, logDF, and logSF provided the best fit. It has been shown that the power of the Mantel test to detect a phylogenetic signal is increased by using Euclidean trait distances and Abouheif’s proximities [40], which may explain why the results obtained for Moran’s and Mantel tests were more congruent when using Abouheif’s proximities. Thus, on the entire sample, we detected a robust phylogenetic signal in a variety of acoustic parameters, even after removing the confounding effect of body size.

**Table 1:** Phylogenetic signal analysis (12S mtDNA) on size-corrected residuals

	**All species**	***Bufo***	**Hylinae**	***Leptodactylus***	***Rana***
	**(90)**	**(32)**	**(32)**	**(12)**	**(14)**
Moran’s I, pairwise distances					
CVA	18.01***	4.60**	2.89*	−0.21	1.11
logDF	16.89***	1.87	4.47**	1.07	3.09*
logSF	4.29**	2.73*	1.71	0.91	−0.04
logSI	8.59***	0.71	5.92***	0.35	0.06
logTON	14.61***	2.82*	2.87*	0.11	1.32
Moran’s I, Abouheif’s proximities					
CVA	8.35***	3.51***	2.45**	0.93	1.12
logDF	7.62***	1.30	3.13**	0.99	2.84**
logSF	5.78***	1.91*	0.97	2.18*	0.19
logSI	4.66***	−0.21	3.04**	0.05	0.26
logTON	5.82***	−0.16	1.60	1.78*	1.08
Blomberg’s *K*, variance-covariance matrix under BM					
CVA	0.346***	0.545**	0.595**	0.507	0.504
logDF	0.362***	0.442**	0.551*	0.930*	0.697*
logSF	0.222***	0.420*	0.382	0.700	0.321
logSI	0.063	0.110	0.497	0.480	0.490
logTON	0.250***	0.405*	0.425	0.824	0.848*
Mantel r, pairwise distances					
logDF	0.335***	0.017	0.131*	0.288	0.096
Mantel r, Abouheif’s proximities					
CVA, logDF, logSF	0.171***	0.164***	0.111***	0.238*	0.223*

*Note:* All analyses were conducted on the phylogenetically corrected and size-corrected residuals of the acoustic parameters [34]. One-tailed tests were conducted for all analyses, under the assumption that species that are genetically more related show more similar values for the acoustic parameters. Significance was estimated using a Monte-Carlo procedure, with 10,000 permutations. For Moran’s I, the values given are the standard deviates of Moran’s I statistic. Mantel r values correspond to the best-fitting model (which can be a multivariate trait distance) obtained using a stepwise forward procedure on the entire sample of 90 species. * P < 0.05, ** P < 0.01, *** P < 0.001.

The intra-clade analyses were not as consistent across statistical methods, which may be due to the reduced power associated with smaller sample sizes [31,42] and to the larger influence exerted by local discrepancies between different models of genetic distance. Nevertheless, we obtained several reliable results that did not depend on a particular statistical test or genetic distance model, such as the significant phylogenetic signals observed for CVA and logSF in *Bufo*, for CVA and logDF in Hylinae, and for logDF in *Rana* (the latter was detected by all methods except the Mantel test on pairwise distances). Importantly, Mantel tests on Abouheif’s proximities and the multivariate trait distances computed from CVA, logDF, and logSF yielded significant correlations for all four clades, indicating that a phylogenetic signal associated with this multivariate trait was detected on intra-clade genetic distances in all cases (note that the magnitude of the Mantel coefficient is often comparatively small even when statistically significant [50]).

### Comparison between 12S mtDNA and rhodopsin

In order to assess the robustness of the phylogenetic signal detected in our acoustic parameters for these four clades and to validate our findings by comparing the results obtained on two genes displaying widely different mutation rates, we repeated our analysis on a subset of 49 species for which genetic data was available for both 12S mtDNA and rhodopsin (exon 1). Only three clades were represented: *Bufo*, Hylinae, and *Rana*. Given that rhodopsin is a very conserved gene, a number of closely related species harbored identical sequences for exon 1. The resolution of the phylogenetic tree derived from the rhodopsin sequence data was therefore relatively limited, rendering Blomberg’s *K* unsuitable for this analysis. Moreover, the small sample size led us to select methods that have been shown to be both powerful and relatively unaffected by the accuracy of the phylogenetic tree [40,42]: Moran’s I (pairwise distances and Abouheif’s proximities), and the Mantel test based on Euclidean trait distances and Abouheif’s proximities.

Following the procedure outlined previously, all acoustic parameters (CVA, logDF, logSF, logSI, and logTON) were regressed on logSVL using the PGLS method, both for entire subset of 49 species and for each clade individually in the case of intra-clade analyses.

Table 2 summarizes the results obtained on the size-corrected residuals. Over all 49 species, significant phylogenetic signals were observed for all acoustic parameters except logSF, as determined by Moran’s tests on pairwise distances and on Abouheif’s proximities, as well as by Mantel tests on Abouheif’s proximities, for both 12S and rhodopsin. These results indicate that the phylogenetic signals observed on genetic distances based on 12S were also detectable when using distances based on the rhodopsin gene. Furthermore, Mantel tests revealed that a multivariate trait based on the Euclidean distances computed from logDF and logTON yielded the best fit with Abouheif’s proximities for both 12S and rhodopsin.

**Table 2:** Comparison between 12S mtDNA and Rhodopsin on size-corrected residuals

	**All species**	***Bufo***	**Hylinae**	***Rana***
	**(49)**	**(15)**	**(24)**	**(10)**
Moran’s I, pairwise distances				
CVA	5.07***	1.97*	1.54	−0.25
	3.98**	2.09*	*1.29*	*−0.79*
logDF	11.94***	−1.20	7.46***	2.22*
	11.28***	*0.17*	6.97***	2.58*
logSF	0.76	1.28	1.26	1.40
	*1.21*	*−0.13*	*1.81*	*1.68*
logSI	8.02***	0.58	1.43	−0.06
	6.29***	*0.06*	*1.08*	*−0.91*
logTON	5.56***	0.52	0.03	3.10**
	6.84***	*−0.56*	*0.44*	3.59**
Moran’s I, Abouheif’s proximities				
CVA	2.75**	2.17*	1.39	−0.33
	3.70***	3.61**	*1.40*	*0.00*
logDF	5.80***	−0.83	4.09***	2.04*
	5.09***	*−0.63*	3.99***	*1.16*
logSF	1.11	−0.20	1.00	0.50
	*1.24*	*0.46*	*1.09*	*1.08*
logSI	2.39*	−0.99	1.14	0.20
	2.79**	*1.35*	*−0.14*	*−1.89*
logTON	2.50**	−1.22	0.18	2.20*
	3.46***	*−1.89*	*1.50*	2.37**
Mantel r, Abouheif’s proximities				
logDF, logTON	0.147***	−0.013	0.153**	0.388**
	0.137***	*−0.111*	0.228***	0.288*

*Note:* All analyses were conducted on the phylogenetically corrected and size-corrected residuals of the acoustic parameters [34]. Values obtained for genetic distances models derived from 12S mtDNA sequences are indicated in regular font, whereas values obtained for distance models derived from rhodopsin sequences are indicated in italics. One-tailed tests were conducted for all analyses, under the assumption that species that are genetically more related show more similar values for the acoustic parameters. Significance was estimated using a Monte-Carlo procedure, with 10,000 permutations. For Moran’s I, the values given are the standard deviates of Moran’s I statistic. Mantel r values correspond to the best-fitting model (which can be a multivariate trait distance) obtained using a stepwise forward procedure on the entire sample of 49 species. * P < 0.05, ** P < 0.01, *** P < 0.001.

Results of intra-clade analyses were broadly consistent, with significance tests generally in agreement both between 12S and rhodopsin and between different models of genetic distance (pairwise distance or Abouheif’s proximities). Notably, a few significant intra-clade phylogenetic signals were detected by all tests on both genes, such as for CVA in Bufo and logDF in Hylinae (in line with the results observed with 12S on larger samples for both clades, see Table 1), as well as for logTON in *Rana*. Finally, Mantel tests on Abouheif’s proximities and the multivariate trait distances computed from logDF and logTON revealed significant correlations for Hylinae and *Rana* with both genes.

## Discussion

In this study, we tested for the presence of a phylogenetic signal in five acoustical features derived from recordings of anuran vocalizations. These acoustical parameters were size-regressed using the PGLS method to account for phylogeny [34]. We first examined 90 species from four clades, using three different models of genetic distance based on 12S mtDNA sequences, after confirming that these sequences displayed little or no substitution saturation. A robust phylogenetic signal was detected in at least four acoustical parameters (CVA, logDF, logSF, and logTON) when considering all species. Intra-clade analyses were less consistent but nevertheless yielded multiple reliable results, such as a significant phylogenetic signal in CVA for *Bufo* and Hylinae, in logDF for Hylinae and *Rana*, and in logSF for *Bufo*. Notably, a multivariate trait computed from CVA, logDF, and logSF was significantly correlated with Abouheif’s matrix of phylogenetic proximities in all four clades.

We then investigated a subset of 49 species from three of these clades for which sequences were available for both rhodopsin and 12S mtDNA. Significant phylogenetic signals were observed over the entire subset for CVA, logDF, logSI, and logTON, for both 12S and rhodopsin. Significance tests on intra-clade analyses were generally in agreement between 12S and rhodopsin and between genetic distance models, and a reliable phylogenetic signal was found with both genes in CVA for *Bufo*, logDF for Hylinae and logTON for *Rana*.

Because the acoustic parameters used in this study were chosen on the basis of their ability to classify calls into one of the four clades studied here [23], the divergences between these parameters computed from advertisement calls of anurans belonging to different clades were expected to be greater than those computed from calls of anurans from the same clade. Indeed, among these five acoustic parameters, CVA, DF, and SF were previously found to be optimal in discriminating between the vocalizations of these clades [23], in line with the best-fitting multivariate trait distance obtained here. However, phylogenetic signals in some of these acoustic parameters were also observed at the intra-clade level in several cases, notably for CVA (*Bufo* and Hylinae) and logDF (Hylinae and *Rana*)*.* These results do not follow automatically from our previous acoustic classification model, which only operated between clades, did not take into account body size and furthermore was generated without a priori knowledge of genetic distances [23]. Thus, the significant intra-clade phylogenetic signals we found strongly suggest that phylogenetic proximity is, at least in these clades and for these acoustic features, a powerful and reliable predictor of the degree of acoustic similarity between advertisement calls of anurans.

A possible limitation of the approach followed in the present study is that it is based on only one or two recordings per species. Thus, the values for the acoustic parameters computed from the recordings represent a limited sample from a potentially broad range of values associated with our species. However, this is unlikely to significantly impact the general conclusions of our study, given that it is based on a large database and that it is concerned with detecting phylogenetic signals at the clade level. Additionally, the Mantel test on Abouheif’s proximities, in particular, has been shown to be relatively robust to sparse sampling and phenotypic variation within species [40]. Nevertheless, follow-up studies may address this issue by sampling several recordings per species.

Polyploidy is fairly common in anurans, which can be problematic for phylogenetic analyses because polyploid taxa do not arise by ordinary cladogenesis [51]. Furthermore, in the case of allopolyploidy, mitochondrial sequences, being inherited only from the maternal side, may not accurately reflect overall genetic distance. Because genetic distances were estimated from 12S mitochondrial DNA sequences in the present study, this could affect our analysis. However, only two species from our sample, *Bufo viridis* and *Hyla chrysoscelis*, are known polyploids (see [51], Additional file 1), suggesting that polyploidy had, at most, a limited impact on our findings.

Ryan [5] observed that characters regulated by behavior and physiology, such as call rate or amplitude modulation, are less conservative than characters that would require modifications of vocal morphology, such as some spectral characters. Supporting Ryan’s observation, and in line with our previous study [29], we found that the only parameters that correlated significantly with body size in our sample were DF and TON, spectral features that are likely to be largely determined by vocal morphology [52]. However, after controlling for body size, we found that both spectral features and spectro-temporal features such as amplitude modulation (CVA in our analysis) and spectral flux exhibited reliable phylogenetic signals, suggesting that there is also a tendency toward phylogenetic conservation for acoustic characters whose link to morphology remains unclear (although Martin [52] related patterns of amplitude modulation in *Bufo* to the presence or absence of well-developed arytenoid valves). Furthermore, while body size may impose an indirect evolutionary constraint on acoustic parameters, and especially on spectral features such as DF [2,5,53], our results show that the trait similarity observed for these spectral features is not solely explained by body size similarity in related species. In that respect, Ryan [53] suggested that the evolution of call features such as DF might also be constrained by signaler-receiver interaction. For instance, modifications in the signal may be more likely to evolve in accordance with preexisting biases in the auditory system of the receiver [54]. Thus, constraints at both production and perceptual levels may be operative. Finally, the values of Blomberg’s *K* were lower than 1 for all acoustic parameters and in all clades (see Table 1). Blomberg *et al.*[31] observed that behavioral traits were generally more labile (i.e., characterized by *K* values below 1) than morphological or physiological traits (typically associated with higher *K* values). Our results thus indicate that the strength of the phylogenetic signal detected in our acoustic parameters is closer to that observed for behavioral traits than for morphological traits in Blomberg *et al.*’s study [31].

Given the central role played by advertisement calls in mate recognition and sexual selection in anurans, it is not surprising that bioacoustic features of these calls have been shown to be reliable taxonomical cues at the local species level. However, the rate at which these features evolve can vary between groups [13,53,55] and call evolution is not always closely related to phylogenetic distance. A recent study on African clawed frogs in fact reported very low phylogenetic signal in the underwater calls of this highly-derived clade [7]. Our results here, studying airborne calls, clearly document the general tendency for more closely related species to exhibit more similar acoustic features, supporting the hypothesis that advertisement call acoustics are at least partly shaped by phylogenetic constraints. Of course, traditional research topics such as the potential influence of ecological constraints (e.g. the calling environment or the presence of sympatric species) [56-58], and other selective pressures, including especially sexual selection [14,59,60], on the evolution of anurans’ vocalizations should not be neglected. Nonetheless, our results suggest that phylogenetic constraints establish important and persistent ground rules, shaping the landscape within which adaptive calls evolution occurs. The clear phylogenetic signal uncovered in the present study, across a wide range of anurans with a broad geographical distribution, is consistent with this “phylogenetic constraint” hypothesis.

Because the analyses presented here were conducted using only a few basic parameters that are easily and automatically measured, and are applicable to a very wide range of sounds, the method described here may have broad biological relevance, applicable to many other clades. Our results indicate an robust relationship between acoustic similarity and genetic relatedness, even after partialling out the effect of body size, in a group of non-vocal learners whose vocalizations are fairly diverse, from the quasi-mechanical trills typical of many *Bufo* species to the whistle-like calls of *Leptodactylus*[23]. It remains a topic for further research whether the acoustic parameters used to characterize advertisement calls in the present study would correlate with genetic distances for other anuran clades. Nevertheless, our results highlight the considerable potential of an approach based on versatile, low-level acoustic parameters, rather than handpicked, clade-specific characteristics. One key advantage of our approach is that it tends to be more resistant to over-fitting and does not require extensive parameterization or manual measurement of the acoustic properties of each vocalization, making these methods potentially useful for a wide range of vocalizations and animal species.

## Conclusions

We found robust evidence for the presence of phylogenetic signals in several acoustic parameters derived from advertisement calls in a sample of 90 species representing four clades of anurans with a wide diversity of call acoustics. Moreover, these phylogenetic signals were detected both across the entire sample and, in several cases, within individual clades, using several measures of genetic distance. These results were generally validated on a subset of 49 species using genetic distances derived from both a highly variable mitochondrial region (12S mtDNA) and from a conserved protein-coding nuclear gene (rhodopsin). Because our methodology is based on general acoustic features found in most animal vocalizations, we suggest that the approach implemented here could fruitfully be applied to other vocal taxa.

## Methods

### Recordings and acoustic analysis

Recordings of anuran vocalizations were digitally copied as WAV files from commercially available CDs (see Additional file 1) comprising calls from North, South, and Central America, Europe, South Africa, Japan and Korea. Only advertisement calls representing single males were used. For each CD track, the longest continuous sequence containing only advertisement calls of a single male was extracted using the Praat software, version 5.1.44 [61]. Two separate non-identical recordings (obtained from different CDs and thus presumably different individuals) were used for 41 species. Only one high-quality recording was available for the remaining 49 species.

The acoustic analysis was conducted using the MIR Toolbox 1.3.2 in MATLAB [62]. Acoustic parameters were analyzed using a window of 40 ms with hop-size of 20 ms, according to the method described in [23].

Mean values for each recording were computed over all 40-ms frames for the following acoustic parameters: dominant frequency (DF), coefficient of variation of root-mean-square amplitude (CVA), spectral flux (SF), spectral flatness or tonality (TON), and spectral irregularity (SI). These five parameters were found to exhibit low multicollinearity in a set of 194 recordings that included the 131 recordings used in the current study [23]. For the 41 species for which two recordings were available, the mean values were averaged over both recordings; the mean values computed from a single recording were used for the remaining 49 species. Detailed values for each recording, as well as the mean values for species for which two recordings were available, are given in Additional file 3.

Temperature data were available for some of the recordings and are provided in Additional file 3. These data were sparse and were not analyzed in the present study. In any case, temperature-induced effects on the acoustical properties of calls are presumably relatively small in comparison to taxonomical or ecological influences [63]. Moreover, DF has rarely been shown to be affected by temperature [28].

### Phylogenetic analysis

#### Nucleotide sequence alignment

Our genetic dataset consisted of nucleotide sequences of approximately 930 bp from the 12S rRNA region of the mitochondrial genome (mtDNA) from 90 species belonging to four clades of anurans (*Bufo*, Hylinae, *Leptodactylus*, and *Rana*). As out-group, the same region of the mtDNA in the Siberian Salamander (*Ranodon sibiricus*, Family: Hynobiidae), California Tiger Salamander (*Ambystoma californiens*, Family: Ambystomatidae) and Western Spadefoot Toad (*Pelobates cultripes*, Family: Pelobatidae) was used.

In addition to the 12S mtDNA sequences, we analyzed a second dataset consisting of nucleotide sequences of 312 bp from exon 1 of the rhodopsin gene from 49 species belonging to three clades (*Bufo*, Hylinae and *Rana). P. cultripes* was used as out-group.

All 12S and rhodopsin sequences were obtained from GenBank, using search and extraction tools developed in Biopython [64], and the validity of obtained DNA sequences was checked using BlastSearch (National Center for Biotechnology Information). The nucleotide sequences were aligned in BioEdit software version 7.0 [65].

The Muscle (implemented in the software MEGA) [66], ClustalW2 [67], and Guidance methods [68] were all tested to obtain a reliable multiple sequence alignment. The ClustalW2 method was eventually used for the multiple sequence alignment on the 12S dataset with the following parameters: gap open penalties (GOP) = 10, gap extension penalties (GEP) = 0.2, gap distance = 5, and the UPGMA clustering method in BioEdit software version 7.0 [65]. In the case of the rhodopsin gene, we used the Muscle Codon option, with GOP = −2.9, GEP = 0 and hydrophobicity multiplier = 1.2. Some sites were manually edited to maximize positional homology.

### Phylogenetic tree reconstruction

The program jMODELTEST [69,70] was used to identify the evolutionary models and other parameters. Based on the Akaike Information Criterion (AICc), the GTR+Γ+I model (general time-reversible) [71] with a proportion of invariant sites (28.6% invariant sites) [72] and rates at other sites varying according to a gamma distribution (gamma shape = 0.53, number of discrete gamma categories = 4) best described the 12S sequence data [73,74]. However, the HKY model (Hasegawa-Kishino-Yano) [75] with gamma site heterogeneity model with 4 rate categories for the discrete approximation of the Gamma distribution rates among sites (HKY +Γ) (gamma shape = 0.31) best described the rhodopsin data.

The phylogenetic relationship among various anuran clades was reconstructed using the Monte-Carlo Markov Chain (MCMC) model implemented in BEAST version 1.6.2 [76]. We used a Yule tree prior that assumes a constant (unknown) lineage birth rate for each branch in the tree. This model is suitable for trees describing the phylogenetic relationship among individuals from many different species. Wide uniform prior distributions were used as defaults. A relaxed clock model [77] with uncorrelated lognormal distribution was used to reconstruct the genetic phylogenies. The MCMC model was run for 30,000,000 generations (10,000,000 generations for rhodopsin) with the initial 1,000,000 steps discarded as burn-in. Trees and model parameters were sampled every 1000 steps thereafter. Effective Sample Sizes (ESS) for mean evolutionary rate, population size and posterior likelihood were found to be > 200 for all the models used. Subsequently, a single target tree with maximum clade credibility (MCC) and median node heights from a sample of trees produced by BEAST were constructed using TreeAnnotator v1.6.2 [78]. The initial 1000 trees were discarded as burn-in. Abouheif proximities were computed from the phylogenetic trees using the function ‘proxTips’ in the package ‘adephylo’ in R [79].

### Estimating pairwise genetic distances using maximum likelihood

Pairwise genetic distances for the 12S and rhodopsin nucleotide sequences were computed using the software MEGA [66]. Both transition and transversion nucleotide substitutions were included in calculating the genetic distance. Gaps and missing data were treated with the partial deletion option, and the bootstrap method with 500 replications was employed.

### Nucleotide substitution saturation test

The nucleotide substitution saturation test was conducted on the 12S mtDNA dataset using DAMBE [46,47]. Because the test can only run on 32 taxonomic units and our sample included 90 species, 10,000 replications with random resampling of subsets of 4, 8, 16, and 32 species were conducted, following the methodology described in [47].

### Snout-vent length

SVL values in mm for males, taken from the literature, were used as an estimate of male body size [29]. Additional file 3 provides the median male SVL values for all 90 species included in this study, and Additional file 1 lists the sources used to obtain these data.

### Statistical analysis

The phylogenetically corrected residuals of the acoustic parameters were computed using the ‘phyl_resid.R’ function in R [34]. Following the methodology described in [34], the ‘phyl_resid.R’ function used a variance-covariance matrix representing phylogenetic relatedness under the Brownian trait evolution model, which was computed from the phylogenetic tree using the function ‘vcv.phylo’ in the package ‘ape’ in R [80]. Multivariate trait Euclidean distances were computed on the standardized size-corrected residuals of the acoustic parameters using the ‘dist’ function from the package ‘stats’ in R (standardization was applied to give equal weight to all parameters).

The standard deviate of Moran’s autocorrelation coefficient [37] was computed using the ‘moran.test’ function from package ‘spdep’ in R [81]. Statistical significance was assessed by Monte-Carlo simulations using the ‘moran.mc’ function from the package ‘spdep’. 10,000 permutations were conducted in each case. Geary’s C was computed in the same way using the functions ‘geary.test’ and ‘geary.mc’, respectively, from the package ‘spdep’. To confirm our analyses on Abouheif’s proximities conducted with the function ‘moran.test’, we used the function ‘abouheif.moran’ from the package ‘adephylo’ in R [79], and obtained nearly identical results (within rounding error).

Blomberg’s *K* was computed using the function ‘phylosig’ from the package ‘phytools’ in R [82]. 10,000 permutations were conducted for significance tests.

Mantel tests were conducted using the ‘mantel’ function from the package ‘ecodist’ in R [83]. 10,000 permutations were conducted for significance tests. To determine the best fit for multivariate trait Euclidean distances, a forward stepwise procedure was conducted in which an acoustic parameter was added to the multivariate trait distance model only if the Mantel correlation coefficient obtained on this distance model was significantly higher than the Mantel correlation coefficient obtained on a trait distance model that did not include this parameter. Because there is no formal test of significance to compare two Mantel correlation coefficients, we used confidence intervals as estimated by bootstrapping (the confidence intervals were also estimated with the ‘mantel’ function from the package ‘ecodist’). A given Mantel coefficient ‘A’ was considered as significantly higher than a coefficient ‘B’ when the value of ‘A’ was higher than 95% of the values of ‘B’ obtained on 10,000 bootstrapping iterations. Note that, to avoid over-fitting, the forward stepwise procedure was only conducted on the entire sample (90 species for the data presented in Table 1, and 49 species for the data presented in Table 2) and not on individual clades.

## Competing interests

The authors declare that they have no competing interests.

## Authors’ contributions

BG collected and analyzed acoustic samples, conducted statistical analyses, and co-wrote the paper, EM collected and analyzed genetic sequences and co-wrote the paper, DB collected genetic sequences, TF designed the study and co-wrote the paper. All authors approved the final version of the manuscript.

## Supplementary Material

Additional file 1 List of commercially available CDs providing recordings for the acoustic analysis, and references from which the snout-vent length values were obtained. Click here for file

Additional file 2 Details of the test of saturation in nucleotide substitutions. Click here for file

Additional file 3 **Mean values for each acoustic parameter (including values computed from each recording) and male snout-vent length values for the 90 species included in the analysis.** Temperature data provided when available. Click here for file
