# Supplementary material for: Phylogenetic signal in the acoustic parameters of the advertisement calls of four clades of anurans
Source: BMC Evol Biol. 2013 Jul 1;13:134. doi: 10.1186/1471-2148-13-134 (PMC3703296; doi:10.1186/1471-2148-13-134)
Supplement: Additional file 1 — List of commercially available CDs providing recordings for the acoustic analysis, and references from which the snout-vent length values were obtained. [file 1471-2148-13-134-S1.docx]

**Additional file 1**

**CD recordings used for the acoustic analysis, with abbreviation indicated in brackets.**

Cocroft, R., Morales, V. R., and McDiarmid, R. W. (**2001**). Frogs of Tambopata, Peru (Macaulay Library of Natural Sounds, Ithaca, NY). **[PERU]**

Cornell Laboratory of Ornithology (**2007**). Voices of the night: the calls of the frogs and toads of Eastern North America (Macaulay Library of Natural Sounds, Ithaca, NY).  **[VOTN]**

Davidson, C. (**1996**). Frog and toad calls of the Rocky Mountains (Macaulay Library of Natural Sounds, Ithaca, NY). **[ROCK]**

De la Riva, I., Reiche, S., Köhler, J., Lötters, S., Bosch, J., Mayer, S., Hennessey, A. B., and Padial, J. M. (**2002**). Sounds of frogs and toads of Bolivia. Edited by R. Marquez, I. De la Riva, J. Bosch & E. Matheu (Alosa, Barcelona, Spain). [**BOL1 and BOL2**]

Elliott, L. (**1994**). The calls of frogs and toads (Northwood Press, Minacqua, WI). **[ELLI]**

Elliott, L., Gerhardt, C., and Davidson, C. (**2009**). The frogs and toads of North America: A comprehensive guide to their identification, behavior, and calls (Houghton Mifflin Harcourt, Boston). **[NOAM]**

Ibanez, R., Stanley Rand, A., and Jaramillo, C. A. (**1999**). The amphibians of Barro Colorado Nature Monument, Soberania National Park and adjacent areas (Editorial Mizrachi & Pujol, Panama). **[PANA]**

Kabaya, T., and Maeda, N. (**1994**). Frog-tunes in the countryside (Yama-Kei Publishers, Tokyo, Japan). **[JAPA]**

Kwet, A., and Marquez, R. (**2010**). Sound guide of the calls of frogs and toads from southern Brazil and Uruguay (Alosa, Barcelona, Spain). 2 CDs: Non-hylidae and Hylidae. **[BRA1 and BRA2]**

Marquez, R., and Matheu, E. (**2004**). Sound guide of frogs and toads of Spain and Portugal (Alosa, Barcelona, Spain). **[SPAP]**

Marty, C., and Gaucher, P. (**1999**). Sound guide to the tailless amphibians of French Guiana (Centre bioacoustique alpin, Mens, France). **[GUIA]**

Park, S. R., and Park, D. S. (**2009**). Open up to the amphibian kingdom (Jisung, Seoul, South Korea). **[KORE]**

Passmore, N. I., and Carruthers, V. C. (**1995**). South African frog calls (Southern Book Publishers and Witwatersrand University Press, Johannesburg, South Africa). **[SAFR]**

Rödel, M. O. (**2000**). Herpetofauna of West Africa, Vol. I Amphibians of the West African savanna (Edition Chimaira, Frankfurt, Germany). **[WAFR]**

Steinbach, G., Podloucky, R., and Roché, J. C. (**2001**). Froschkonzert am Gartenreich (Frog concert in the garden) (Franckh-Kosmos Verlags, Stuttgart, Germany). **[EURO]**

**List of references used for information about snout-vent length:**

Amphibiaweb. <http://amphibiaweb.org/index.html> [Accessed Feb 29 2012].

Beebee, T., & Griffiths, R. (2000). *The New Naturalist: Amphibians and reptiles- a natural history of the British herpetofauna*. Harper Collins Publishers: London.

Castellano, S., Rosso, A., Doglio, S., & Giacoma, C. (1999). Body size and calling variation in the green toad (Bufo viridis). *Journal of Zoology* **248**, 83-90.

Clark, H.O., Jr. (2011). Arizona Toad, *Anaxyrus microscaphus* (Cope “1866” 1867). Herpetofauna of the 100-mile Circle. *Sonoran Herpetologist* **24**, 14-15.

De Almeida Prado, C.P., Uetanabaro, M., & Lopes, F.S. (2000). Reproductive Strategies of Leptodactylus chaquensis and L. podicipinus in the Pantanal, Brazil. *Journal of Herpetology* **34**, 135-139.

De-Carvalho, C.B., Freitas, E.B., Faria, R.G., Batista, R.C., Batista, C.C., Coelho, W.A., & Bocchiglieri, A. (2008). Natural history of *Leptodactylus mystacinus* and *Leptodactylus fuscus* (Anura: Leptodactylidae) in the Cerrado of Central Brazil. *Biota Neotropica* **8**, 105-116.

De Sa, R.O. (1996). Hylidae, *Hyla*, *H. multifasciata*. *Catalogue of American Amphibians and Reptiles*, 624.1 – 624.4. Society for the Study of Amphibians and Reptiles.

Duellman, W.E., & Thomas, R. (1996). Anuran Amphibians from a seasonally dry forest in southeastern Peru and comparisons of the anurans among sites in the upper Amazon basin. *Occasional papers of the Natural History Museum* **180**, 1-34. The University of Kansas: Lawrence, Kansas.

Duellman, W.E., De la Riva, I., & Wild, E.R. (1997). Frogs of the *Hyla armata* and *Hyla pulchella* groups in the Andes of South America, with definitions and analyses of phylogenetic relationships of Andean groups of Hyla. *Scientific papers of the Natural History Museum* **3**, 1-41. The University of Kansas: Lawrence, Kansas.

du Preez, L., & Carruthers, V. (2009). *A Complete Guide to the Frogs of Southern Africa*. Struik Nature: South Africa.

Heyer, W.R. (1978). Systematics of the Fuscus group of the frog genus Leptodactylus (Amphibia, Leptodactylidae). *Natural History Museum of Los Angeles Country: Science Bulletin* **29**, 1-84.

Heyer, W.R. (1995). South American rocky habitat *Leptodactylus* (Amphibia:Anura:Leptodactylidae) with description of two new species. *Proceedings of the Biological Society of Washington* **108**, 695-716.

Hoedl, W. (1977). Call differences and calling site segregation in Anuran species from Central Amazonian Floating Meadows. *Oecologia* **28**, 351-363.

Köhler, J., & Lötters, S (1999). Annotated list of amphibia records from the Departamento Pando, Bolivia, with description of some advertisement calls. Bonner zoologische Beiträge **48**, 259-273.

Krone, A., & Kitzmann, B. (2008). Zur Habitatwahl des Moorfrosches (Rana arvalis) im urbanen Raum, am Beispiel einer Berliner Population. In *Der Moorfrosch/The Moor Frog*, D. Glandt, & R. Jehle (eds.).  *Zeitschrift für Feldherpetologie*, Supplement 13: 467–476.

Lima, A. P. Magnusson, W.E., Menin, M., Erdtmann, L. K., Rodrigues, D.J., Keller, C., & Hödl, W. (2006). *Guide to the Frogs of Reserva Adolpho Ducke (Central Amazonia)*. Manaus: Attema.

Marquez, R., De la Riva, I., & Bosch, J. (1993). Advertisement Calls of Bolivian Species of Hyla (Amphibia, Anura, Hylidae). *Biotropica* **25**, 426-443.

Reaser, J.K. (2000). Demographic analysis of the Columbia spotted frog (Rana luteiventris): Case study in spatiotemporal variation. *Canadian Journal of Zoology* **78**, 1158–1167.

Ritke, M.E., Babb, J.G., & Ritke, M.K. (1990). Life history of the Gray Treefrog (*Hyla chrysoscelis*) in Western Tennessee. *Journal of Herpetology* **24**, 135-141.

Rodriguez, L.O., & Duellman, W.E. (1994). *Guide to the Frogs of the Iquitos Region, Amazonia Peru*. Natural History Museum, Kansas University: Lawrence, Kansas.

Ryser, J. (1988). Determination of growth and maturation in the common frog, *Rana temporaria*, by skeletochronology. *Journal of Zoology* **216**, 673-685.

Savage, J.M. (2002). *The Amphibians and Reptiles of Costa Rica*. Chicago: University of Chicago Press.

Schwartz, A., & Harrison, J.R. (1956). A new subspecies of Gopher Frog (*Rana capito* Leconte). *Proceedings of the Biological Society of Washington* **69**, 135-144.

Sullivan, B. K. (1986). Advertisement call variation in the Arizona Tree frog, *Hyla wrightorum* Taylor, 1938. *Great Basin Naturalist* **46**, 378-381.

Woodland Park Zoo. <http://www.zoo.org/animal-facts/smooth-sided-toad> [Accessed Feb 29 2012].

Wright, A.A., & Wright, A.H. (1933). *The Frogs and Toads of the United States and Canada*. Comstock Publishing: United States.
