# Supplementary material for: Phylogenetic signal in the acoustic parameters of the advertisement calls of four clades of anurans
Source: BMC Evol Biol. 2013 Jul 1;13:134. doi: 10.1186/1471-2148-13-134 (PMC3703296; doi:10.1186/1471-2148-13-134)
Supplement: Additional file 2 — Details of the test of saturation in nucleotide substitutions. [file 1471-2148-13-134-S2.docx]

**Additional file 2**

**Results of the nucleotide substitution saturation test (DAMBE) on the 12S mtDNA dataset**

The nucleotide substitution saturation test calculates an index of substitution saturation (Iss), which is compared to a critical value which is computed for a symmetrical (Iss.cSym) or extremely asymmetrical (Iss.cAsym) tree topology. Because the test can only run on 32 taxonomic units and our sample included 90 species, 10,000 replications with random resampling of subsets of 4, 8, 16, and 32 species were conducted.

**1. Analysis performed on fully resolved sites:**

| Num OTU | Iss | Iss.cSym | T | DF | P | Iss.cAsym | T | DF | P |
| --- | --- | --- | --- | --- | --- | --- | --- | --- | --- |
| 4 | 0.156 | 0.800 | 39.069 | 571 | < 0.0001 | 0.768 | 37.132 | 571 | < 0.0001 |
| 8 | 0.153 | 0.758 | 34.214 | 571 | < 0.0001 | 0.648 | 27.952 | 571 | < 0.0001 |
| 16 | 0.155 | 0.733 | 31.915 | 571 | < 0.0001 | 0.522 | 20.286 | 571 | < 0.0001 |
| 32 | 0.158 | 0.710 | 30.277 | 571 | < 0.0001 | 0.382 | 12.283 | 571 | < 0.0001 |

Note: NumOTU: number of operational taxonomic units. Iss: index of substitution saturation. Iss.cSym: critical value for symmetrical tree topology. Iss.cAsym: critical value for extremely assymetrical tree topology. T: T value. DF: degrees of freedom. P: probability that Iss is significantly different from the critical value (Iss.cSym or Iss.cAsym). Two-tailed tests were used. Results based on 10,000 replications.

**2. Analysis performed on all sites:**

| Num OTU | Iss | Iss.cSym | T | DF | P | Iss.cAsym | T | DF | P |
| --- | --- | --- | --- | --- | --- | --- | --- | --- | --- |
| 4 | 0.378 | 0.821 | 20.949 | 1017 | < 0.0001 | 0.789 | 19.436 | 1017 | < 0.0001 |
| 8 | 0.402 | 0.789 | 13.824 | 1017 | < 0.0001 | 0.684 | 10.056 | 1017 | < 0.0001 |
| 16 | 0.442 | 0.772 | 9.453 | 1017 | < 0.0001 | 0.575 | 3.807 | 1017 | 0.0001 |
| 32 | 0.490 | 0.749 | 6.152 | 1017 | < 0.0001 | 0.445 | 1.078 | 1017 | 0.2814 |

Note: NumOTU: number of operational taxonomic units. Iss: index of substitution saturation. Iss.cSym: critical value for symmetrical tree topology. Iss.cAsym: critical value for extremely assymetrical tree topology. T: T value. DF: degrees of freedom. P: probability that Iss is significantly different from the critical value (Iss.cSym or Iss.cAsym). Two-tailed tests were used. Results based on 10,000 replications.

**References:**

Xia X, Xie Z, Salemi M, Chen L, Wang Y: **An index of substitution saturation and its application**. *Mol. Phylogenet. Evol.* 2003, **26**:1–7.

Xia X, Lemey P: **Assessing substitution saturation with DAMBE**. In *The Phylogenetic Handbook: A Practical Approach to DNA and Protein Phylogeny*. 2nd edition. edited by Lemey P, Salemi M, Vandamme A-M Cambridge: Cambridge University Press; 2009:615–630.
