# Supplementary material for: Phylogenetic signal in the acoustic parameters of the advertisement calls of four clades of anurans
Source: BMC Evol Biol. 2013 Jul 1;13:134. doi: 10.1186/1471-2148-13-134 (PMC3703296; doi:10.1186/1471-2148-13-134)
Supplement: Additional file 3 — Mean values for each acoustic parameter (including values computed from each recording) and male snout-vent length values for the 90 species included in the analysis. Temperature data provided when available. [file 1471-2148-13-134-S3.docx]

**Additional file 3: Snout-vent length, acoustic parameters and recording information for the 90 species included in the analysis.**

| Scientific name | SVL (mm) |  |  |  |  |  |  |  |  |
| --- | --- | --- | --- | --- | --- | --- | --- | --- | --- |
|  |  | DF (Hz) | CVA | SF | TON (×10^-5^) | SI | CD abbr. | Track No. | TEMP (°C) |
| ***Bufo:*** |  |  |  |  |  |  |  |  |  |
| *Bufo alvarius* | 118 | 1155 | 35.6 | 69.2 | 41.4 | 0.581 | NOAM | 46 | 26 |
| *Bufo americanus**  Recording 1:  Recording 2: | 70 | 1504  1570  1439 | 19.7  16.1  23.4 | 95.3  138.5  52.0 | 24.6  32.8  16.3 | 0.979  0.485  1.475 | NOAM  VOTN | 31  57 | 18  N/A |
| *Bufo baxteri* | 54 | 1636 | 23.2 | 44.7 | 6.4 | 0.909 | NOAM | 42 | 19 |
| *Bufo boreas* | 82 | 1231 | 42.4 | 64.7 | 28.0 | 0.949 | NOAM | 48 | N/A |
| *Bufo bufo**  Recording 1:  Recording 2: | 55 | 846  949  743 | 49.7  48.7  50.8 | 123.9  131.7  116.0 | 5.5  7.6  3.4 | 0.553  0.403  0.703 | EURO  SPAP | 05  36 | N/A N/A |
| *Bufo californicus* | 66 | 1540 | 14.8 | 34.7 | 5.1 | 1.440 | NOAM | 51 | 19 |
| *Bufo castaneoticus* | 37 | 1633 | 32.3 | 67.0 | 60.9 | 0.273 | BOL1 | 03 | 26 |
| *Bufo cognatus**  Recording 1:  Recording 2: | 71 | 2133  2075  2190 | 28.8  34.1  23.4 | 44.6  44.2  45.1 | 51.1  33.8  68.4 | 0.491  0.618  0.364 | NOAM  ROCK | 40  25 | 17 21 |
| *Bufo coniferus* | 63 | 903 | 20.0 | 70.9 | 33.7 | 1.119 | PANA | 04 | N/A |
| *Bufo debilis**  Recording 1:  Recording 2: | 34 | 3253  3346  3160 | 10.2  10.6  9.8 | 40.4  48.6  32.2 | 12.5  12.6  12.4 | 0.804  0.857  0.751 | NOAM  ROCK | 44  31 | 23  18 |
| *Bufo fowleri**  Recording 1:  Recording 2: | 63 | 1874  1768  1981 | 24.1  17.8  30.3 | 54.4  49.3  59.5 | 16.6  3.5  29.6 | 0.504  0.602  0.406 | NOAM  VOTN | 32  63 | 20  N/A |
| *Bufo gariepensis* | 62 | 1488 | 39.3 | 44.0 | 7.4 | 0.484 | SAFR | 02 | N/A |
| *Bufo garmani* | 75 | 866 | 36.1 | 39.5 | 6.5 | 0.354 | SAFR | 02 | N/A |
| *Bufo granulosus**  Recording 1:  Recording 2: | 65 | 2823  2697  2950 | 23.0  32.0  14.1 | 49.3  46.0  52.6 | 26.5  11.6  41.3 | 0.628  0.481  0.774 | GUIA  BOL1 | 05  05 | N/A  N/A |
| *Bufo guttatus* | 140 | 1080 | 56.7 | 74.6 | 8.9 | 0.549 | GUIA | 06 | N/A |
| *Bufo gutturalis* | 79 | 1354 | 34.8 | 33.8 | 201.4 | 0.631 | SAFR | 02 | N/A |
| *Bufo haematiticus* | 52 | 2283 | 39.8 | 310.0 | 280.9 | 0.860 | PANA | 06 | N/A |
| *Bufo hemiophrys**  Recording 1: Recording 2: | 62 | 1570  1502  1637 | 19.9  16.5  23.3 | 41.7  54.9  28.5 | 5.5  4.6  6.5 | 0.940  0.946  0.934 | NOAM  ROCK | 41  29 | 7  9 |
| *Bufo hustonensis* | 58 | 2076 | 7.6 | 95.3 | 20.5 | 0.275 | NOAM | 38 | 16 |
| *Bufo japonicus* | 110 | 823 | 34.1 | 88.3 | 264.6 | 0.832 | JAPA | 40 | N/A |
| *Bufo maculatus**  Recording 1: Recording 2: | 52 | 1833  1583  2082 | 42.6  32.8  52.4 | 42.8  57.1  28.5 | 530.2  6.3  1054.1 | 0.589  0.659  0.519 | SAFR  WAFR | 02  N/A | N/A N/A |
| *Bufo marinus**  Recording 1: Recording 2: | 116 | 614  608  619 | 30.9  23.6  38.2 | 143.4  163.5  123.4 | 46.6  34.3  59.0 | 0.654  0.657  0.652 | PANA  NOAM | 02  39 | N/A 27 |
| *Bufo microscaphus* | 66 | 1201 | 20.4 | 40.5 | 6.5 | 1.057 | NOAM | 47 | 16 |
| *Bufo nebulifer* | 76 | 1360 | 17.7 | 102.9 | 18.7 | 0.518 | NOAM | 36 | 19 |
| *Bufo punctatus* | 54 | 2408 | 24.0 | 8.0 | 4.0 | 1.356 | NOAM | 43 | 17 |
| *Bufo quercicus**  Recording 1: Recording 2: | 25 | 4386  4382  4391 | 36.8  35.8  37.7 | 141.4  180.8  102.1 | 18.8  10.3  27.3 | 0.929  0.686  1.172 | NOAM  ELLI | 35  38 | 25  N/A |
| *Bufo retiformis**  Recording 1: Recording 2: | 44 | 3101  3253  2949 | 20.8  22.2  19.5 | 32.2  43.7  20.8 | 61.3  18.8  103.9 | 0.349  0.269  0.430 | NOAM  ROCK | 45  33 | N/A  27 |
| *Bufo schneideri**  Recording 1: Recording 2: | 136 | 684  717  652 | 38.4  38.7  38.1 | 157.2  160.6  153.8 | 145.0  162.2  127.8 | 0.654  0.623  0.685 | BOL1  BRA2 | 07  05 | 25 16 |
| *Bufo speciosus* | 71 | 2501 | 18.1 | 59.7 | 12.7 | 0.581 | NOAM | 37 | 17 |
| *Bufo vertebralis* | 26 | 2587 | 22.0 | 42.3 | 38.9 | 1.001 | SAFR | 02 | N/A |
| *Bufo viridis**  Recording 1: Recording 2: | 67 | 1372  1507  1236 | 33.9  26.8  41.0 | 108.0  79.4  136.6 | 21.1  12.5  29.6 | 0.576  0.858  0.294 | EURO  SPAP | 07  45 | N/A  N/A |
| *Bufo woodhousii**  Recording 1: Recording 2: | 78 | 1433  1420  1445 | 19.2  15.4  23.1 | 27.3  36.93  17.71 | 65.0  5.6  124.4 | 0.742  0.848  0.636 | NOAM  ROCK | 33  19 | N/A  N/A |
| **Hylinae:** |  |  |  |  |  |  |  |  |  |
| *Dendropsophus allenorum* | 19 | 4725 | 54.7 | 25.8 | 760.1 | 0.615 | PERU | 11 | 25 |
| *Dendropsophus bifurcus* | 26 | 3018 | 52.5 | 67.8 | 90.9 | 0.546 | BOL1 | 31 | 27 |
| *Dendropsophus brevifrons* | 19 | 3686 | 52.2 | 133.9 | 89.5 | 0.834 | GUIA | 21 | N/A |
| *Dendropsophus ebraccatus* | 25 | 3351 | 43.4 | 51.8 | 801.1 | 1.006 | PANA | 25 | N/A |
| *Dendropsophus koechlini* | 21 | 4670 | 51.4 | 32.2 | 371.4 | 1.105 | PERU | 17 | 23 |
| *Dendropsophus leucophyllatus**  Recording 1: Recording 2: | 35 | 3462  2813  4110 | 48.3  42.7  54.0 | 80.6  109.8  51.9 | 144.5  165.2  123.7 | 1.022  1.046  0.999 | BOL1  PERU | 45  20 | N/A  N/A |
| *Dendropsophus microcephalus* | 22 | 5525 | 22.7 | 116.1 | 567.7 | 0.947 | PANA | 27 | N/A |
| *Dendropsophus nanus**  Recording 1: Recording 2: | 20 | 4213  4354  4072 | 50.8  65.3  36.3 | 71.2  73.2  69.2 | 403.9  113.6  694.1 | 0.727  0.627  0.828 | GUIA  BRA1 | 30  08 | N/A  N/A |
| *Dendropsophus parviceps* | 17 | 3879 | 51.8 | 103.4 | 177.9 | 0.743 | BOL1 | 54 | 24 |
| *Dendropsophus rhodopeplus* | 22 | 7214 | 54.6 | 61.7 | 420.8 | 0.944 | PERU | 24 | 22 |
| *Dendropsophus riveroi**  Recording 1: Recording 2: | 20 | 5307  5130  5484 | 50.0  34.5  65.4 | 107.0  166.3  47.6 | 711.1  953.8  468.6 | 0.933  0.970  0.896 | BOL1  PERU | 61  25 | 22  25 |
| *Dendropsophus sarayacuensis**  Recording 1: Recording 2: | 27 | 3029  2443  3614 | 52.3  55.2  49.5 | 72.4  49.3  95.4 | 280.6  331.3  230.0 | 0.866  0.990  0.741 | PERU  BOL1 | 26  62 | 24  24 |
| *Dendropsophus triangulum* | 26 | 6096 | 69.7 | 37.3 | 530.7 | 0.409 | PERU | 27 | 25 |
| *Hyla andersonii**  Recording 1: Recording 2: | 35 | 1515  1142  1887 | 36.3  34.5  38.0 | 61.3  65.2  57.4 | 41.8  18.4  65.2 | 0.672  0.634  0.710 | NOAM  ELLI | 03  16 | N/A  N/A |
| *Hyla arenicolor* | 41 | 1608 | 29.8 | 56.7 | 53.4 | 0.591 | NOAM | 10 | 22 |
| *Hyla avivoca**  Recording 1: Recording 2: | 34 | 2293  2381  2205 | 48.1  55.0  41.2 | 74.6  60.5  88.6 | 66.7  58.9  74.5 | 0.632  0.532  0.733 | NOAM  ELLI | 08  22 | 24  N/A |
| *Hyla chrysoscelis**  Recording 1: Recording 2: | 46 | 2295  2228  2363 | 23.4  28.1  18.6 | 23.1  22.9  23.3 | 54.3  12.8  95.8 | 0.802  0.796  0.807 | NOAM  ELLI | 07  21 | 20  N/A |
| *Hyla japonica**  Recording 1: Recording 2: | 31 | 2773  2624  2923 | 44.9  22.9  66.8 | 89.4  159.3  19.4 | 196.4  200.7  192.0 | 0.875  1.115  0.635 | KORE  JAPA | 08  07 | N/A  N/A |
| *Hyla squirella**  Recording 1: Recording 2: | 30 | 3061  3191  2930 | 47.7  37.7  57.7 | 51.8  56.7  47.0 | 218.4  94.0  342.8 | 0.539  0.547  0.531 | NOAM  ELLI | 04  18 | 24  N/A |
| *Hyla wrightorum* | 41 | 1993 | 38.6 | 31.3 | 16.0 | 0.486 | NOAM | 09 | 17 |
| *Hyloscirtus charazani* | 70 | 1580 | 43.3 | 74.0 | 255.0 | 0.571 | BOL1 | 33 | 12 |
| *Hypsiboas albopunctatus**  Recording 1: Recording 2: | 53 | 2452  3028  1876 | 44.1  42.7  45.6 | 84.3  76.6  92.1 | 271.8  256.2  287.4 | 0.593  0.588  0.599 | BOL1  BRA1 | 24  13 | N/A  22 |
| *Hypsiboas boans**  Recording 1: Recording 2: | 101 | 963  983  943 | 50.4  56.6  44.1 | 61.5  54.6  68.4 | 146.7  18.5  274.8 | 0.286  0.303  0.269 | GUIA  PANA | 20  22 | N/A  N/A |
| *Hypsiboas fasciatus**  Recording 1: Recording 2: | 38 | 2361  2157  2564 | 51.3  43.9  58.8 | 50.7  68.2  33.2 | 653.6  338.6  968.6 | 0.657  0.455  0.859 | BOL1  PERU | 35  14 | N/A  24 |
| *Hypsiboas geographicus**  Recording 1: Recording 2: | 51 | 892  832  951 | 51.4  51.4  51.4 | 78.4  69.4  87.4 | 68.2  50.9  85.4 | 0.394  0.432  0.356 | GUIA  BOL1 | 24  37 | N/A  N/A |
| *Hypsiboas marianitae* | 48 | 955 | 51.7 | 38.4 | 149.1 | 0.578 | BOL1 | 47 | 18 |
| *Hypsiboas multifasciatus* | 48 | 1719 | 50.8 | 78.8 | 38.3 | 0.438 | GUIA | 29 | N/A |
| *Hypsiboas punctatus* | 31 | 1697 | 26.6 | 110.0 | 122.1 | 0.443 | GUIA | 32 | N/A |
| *Hypsiboas raniceps**  Recording 1: Recording 2: | 49 | 2181  2130  2233 | 46.6  40.2  53.1 | 61.6  69.9  53.3 | 224.2  58.0  390.3 | 0.641  0.562  0.719 | GUIA  BOL1 | 33  56 | N/A  N/A |
| *Hypsiboas riojanus* | 47 | 2191 | 48.3 | 108.8 | 90.7 | 0.420 | BOL1 | 28 | 14 |
| *Hypsiboas rosenbergi* | 70 | 525 | 33.2 | 122.7 | 390.3 | 0.531 | PANA | 23 | N/A |
| *Hypsiboas rufitelus* | 44 | 2634 | 63.3 | 112.8 | 438.3 | 0.563 | PANA | 21 | N/A |
| ***Leptodactylus:*** |  |  |  |  |  |  |  |  |  |
| *Leptodactylus bolivianus* | 73 | 680 | 56.3 | 220.2 | 94.1 | 1.103 | PANA | 46 | N/A |
| *Leptodactylus bufonius* | 52 | 1678 | 34.0 | 372.6 | 17.4 | 0.607 | BOL2 | 08 | N/A |
| *Leptodactylus chaquensis* | 71 | 720 | 37.8 | 98.2 | 24.5 | 0.573 | BRA1 | 50 | 18 |
| *Leptodactylus didymus**  Recording 1: Recording 2: | 55 | 904  968  839 | 49.3  46.9  51.6 | 179.1  161.9  196.3 | 74.5  135.1  13.9 | 0.981  0.931  1.031 | PERU  BOL2 | 58  11 | 24  27 |
| *Leptodactylus elenae* | 43 | 1397 | 52.7 | 239.7 | 141.6 | 1.005 | BOL2 | 12 | N/A |
| *Leptodactylus fuscus**  Recording 1: Recording 2: | 43 | 2167  2172  2161 | 38.0  38.0  38.0 | 285.1  255.3  314.9 | 39.0  39.3  38.7 | 1.272  1.250  1.295 | GUIA  PANA | 64  48 | N/A  N/A |
| *Leptodactylus knudseni**  Recording 1: Recording 2: | 131 | 485  482  488 | 37.3  38.8  35.7 | 121.7  161.9  81.4 | 56.9  5.5  108.2 | 0.797  0.865  0.729 | BOL2  PERU | 16  59 | N/A  23 |
| *Leptodactylus leptodactyloides* | 41.6 | 1076 | 24.5 | 109.5 | 278.0 | 0.668 | PERU | 60 | 23 |
| *Leptodactylus notoaktites* | 47 | 1192 | 41.2 | 168.1 | 78.8 | 0.430 | BRA1 | 48 | 26 |
| *Leptodactylus pentadactylus**  Recording 1: Recording 2: | 155 | 698  479  917 | 45.4  43.6  47.1 | 126.7  143.6  109.9 | 23.1  34.4  12.0 | 0.723  1.089  0.357 | PANA  PERU | 45  61 | N/A  24 |
| *Leptodactylus podicipinus* | 35 | 1728 | 29.1 | 178.8 | 124.6 | 1.024 | BOL2 | 21 | N/A |
| *Leptodactylus rhodomystax**  Recording 1: Recording 2: | 84 | 2021  2133  1909 | 43.6  41.1  46.1 | 138.3  173.1  103.5 | 49.9  6.4  93.3 | 0.628  0.581  0.675 | GUIA  BOL2 | 70  22 | N/A  24 |
| ***Rana*:** |  |  |  |  |  |  |  |  |  |
| *Rana capito**  Recording 1: Recording 2: | 84 | 505  367  642 | 41.2  38.2  44.1 | 65.9  64.8  67.1 | 111.0  35.3  186.7 | 0.532  0.623  0.441 | NOAM  ELLI | 62  12 | N/A  N/A |
| *Rana catesbeiana**  Recording 1: Recording 2: | 133 | 191  178  205 | 39.5  39.2  39.8 | 23.4  29.6  17.3 | 28.2  14.7  41.9 | 0.329  0.375  0.283 | NOAM  ELLI | 53  01 | N/A  N/A |
| *Rana clamitans**  Recording 1: Recording 2: | 62 | 394  415  372 | 47.6  46.3  48.9 | 27.4  34.2  20.5 | 62.9  53.7  72.2 | 0.379  0.359  0.400 | NOAM  ROCK | 54  73 | N/A  N/A |
| *Rana grylio**  Recording 1: Recording 2: | 117 | 263  261  266 | 34.0  31.5  36.5 | 63.0  42.3  83.8 | 36.6  7.8  65.4 | 0.826  0.467  1.184 | NOAM  VOTN | 55  88 | N/A  N/A |
| *Rana heckscheri**  Recording 1: Recording 2: | 107 | 359  292  426 | 34.6  36.7  32.4 | 21.5  23.0  20.0 | 54.8  24.0  85.6 | 0.569  0.606  0.532 | NOAM  ELLI | 60  14 | 24  N/A |
| *Rana ishikawae* | 97 | 1194 | 51.5 | 45.5 | 21.9 | 0.673 | JAPA | 34 | N/A |
| *Rana japonica* | 48 | 1430 | 67.3 | 50.6 | 72.2 | 0.270 | JAPA | 19 | N/A |
| *Rana luteiventris* | 54 | 637 | 46.4 | 34.5 | 285.6 | 0.494 | NOAM | 77 | 6 |
| *Rana muscosa* | 56 | 1041 | 40.1 | 38.3 | 277.7 | 0.401 | NOAM | 79 | 18 |
| *Rana nigromaculata**  Recording 1: Recording 2: | 70 | 1534  955  2113 | 47.5  47.3  47.8 | 74.3  137.1  11.4 | 218.0  84.3  351.8 | 0.411  0.436  0.387 | KORE  JAPA | 07  27 | N/A  N/A |
| *Rana pipiens**  Recording 1: Recording 2: | 66 | 737  746  728 | 56.1  68.5  43.6 | 39.6  28.3  50.8 | 168.2  51.5  285.0 | 0.460  0.284  0.636 | NOAM  ELLI | 65  10 | 21  N/A |
| *Rana plancyi* | 50 | 1104 | 41.7 | 158.5 | 441.2 | 0.513 | KORE | 11 | N/A |
| *Rana rugosa* | 40 | 1236 | 35.1 | 73.4 | 697.4 | 0.349 | KORE | 13 | N/A |
| *Rana temporaria**  Recording 1: Recording 2: | 75 | 408  386  430 | 52.3  54.3  50.2 | 113.6  105.0  122.2 | 81.6  92.8  70.5 | 0.439  0.452  0.426 | EURO  SPAP | 01  67 | N/A  N/A |

Note: For the species names followed by an asterisk (*), the mean values for the acoustic parameters, averaged from two recordings for these species, are provided along with the values computed from each recording. SVL: snout-vent length. DF: dominant frequency. CVA: coefficient of variation of the root-mean-square amplitude. SF: spectral flux. TON: tonality or spectral flatness. SI: spectral irregularity. TEMP: temperature at the time and place of recording, as provided by the CD producers. The CD abbreviations are listed in Additional file 1.
